# Polyester type polyHIPE scaffolds with an interconnected porous structure for cartilage regeneration

**DOI:** 10.1038/srep28695

**Published:** 2016-06-24

**Authors:** Jakob Naranda, Maja Sušec, Uroš Maver, Lidija Gradišnik, Mario Gorenjak, Andreja Vukasović, Alan Ivković, Marjan Slak Rupnik, Matjaž Vogrin, Peter Krajnc

**Affiliations:** 1 University Clinical Centre Maribor, Department of Orthopaedics, Ljubljanska ulica 5, SI-2000 Maribor, Slovenia; 2 University of Maribor, Faculty of Chemistry and Chemical Engineering, PolyOrgLab, Smetanova 17, SI-2000 Maribor, Slovenia; 3 Polymer Technology College, Ozare 19, SI-2380 Slovenj Gradec, Slovenia; 4 University of Maribor, Faculty of Medicine, Institute of Biomedical Sciences, Taborska ulica 8, SI-2000 Maribor, Slovenia; 5 University of Zagreb, School of Medicine, Department for Histology and Embryology, Salata 3, 10000 Zagreb, Croatia; 6 Department of Orthopaedic Surgery, University Hospital Sveti Duh, Sveti Duh 64, 10000 Zagreb, Croatia; 7 Medical University of Vienna, Center for Physiology and Pharmacology, Schwarzspanierstrasse 17, 1090 Vienna, Austria

## Abstract

Development of artificial materials for the facilitation of cartilage regeneration remains an important challenge in orthopedic practice. Our study investigates the potential for neocartilage formation within a synthetic polyester scaffold based on the polymerization of high internal phase emulsions. The fabrication of polyHIPE polymer (PHP) was specifically tailored to produce a highly porous (85%) structure with the primary pore size in the range of 50–170 μm for cartilage tissue engineering. The resulting PHP scaffold was proven biocompatible with human articular chondrocytes and viable cells were observed within the materials as evaluated using the Live/Dead assay and histological analysis. Chondrocytes with round nuclei were organized into multicellular layers on the PHP surface and were observed to grow approximately 300 μm into the scaffold interior. The accumulation of collagen type 2 was detected using immunohistochemistry and chondrogenic specific genes were expressed with favorable collagen type 2 to 1 ratio. In addition, PHP samples are biodegradable and their baseline mechanical properties are similar to those of native cartilage, which enhance chondrocyte cell growth and proliferation.

Cartilage tissue engineering remains a field with possibilities for improvement despite very intense research efforts in recent times^1^,^2^,^3^,^4^. Scaffolds used for this purpose need to be engineered 3-D matrices that act as an initial support for the desired cells to attach, proliferate and form their native extracellular matrix (ECM)^5^,^6^,^7^,^8^. Since the microstructure of the scaffolds used in cartilage tissue engineering (e.g. pore shape, size, porosity and interconnectivity) can directly affect the behavior of the seeded cells and is usually associated with the mechanical properties, a variety of materials were extensively studied to control the mentioned scaffold characteristics^9^,^10^,^11^,^12^,^13^,^14^,^15^,^16^. The ultimate goal of cartilage tissue engineering is the creation of artificial matrices that can be used in *in vivo* application (e.g. in cartilage joint repair). Therefore developed matrices should be biocompatible, biodegradable and readily assessable with the ability of the construction of pores of different shapes and sizes. Furthermore, they should be capable to integrate well with normal articular cartilage and subchondral bone, as well as possess and maintain the mechanical properties of native cartilage tissue^1^,^2^,^3^,^4^,^5^,^6^. Finally, scaffolds used in cartilage tissue engineering should enhance chondrocyte differentiation, promote production of cartilage specific ECM (e.g. aggrecan and collagen type 2) and preserve the desired cellular morphology^17^,^18^,^19^. Failure to ensure the mentioned leads to dedifferentiation and altered gene expression patterns from chondrocyte-specific to (mostly) fibroblastic or chondroprogenitor-like cells^1^,^2^,^20^.

An interesting approach to scaffold formation presents the so-called emulsion templating technique, which was already applied to produce hierarchically porous materials using a series of different monomers and polymerization mechanisms^21^,^22^. Variation of the latter can lead to a range of functionalities and possible applications of the produced materials. The resulting material; termed polyHIPE (PHP), is a synthetic highly porous material prepared by the polymerization of high internal phase emulsions (HIPEs)^23^. The advantages of PHP compared to other foam-like synthetic materials are in its highly flexible topological characteristics enabling production of PHPs with different porosities (74–99%), pore sizes (1–300 μm), interconnecting pores (0.1–20 μm) and compressive moduli up to 60 MPa^24^,^25^,^26^. These characteristics can also be adjusted to design a scaffold suitable for cartilage tissue engineering by altering the manufacturing procedure (e.g. optimized stirring rate, mixing time and temperature) while retaining its uniform structure, mechanical properties and biodegradability. The materials used in PHP preparation and their byproducts were proven non-cytotoxic and different PHP variations have been considered as scaffolds for biological cell growth including human fibroblasts^27^, osteoblasts and bone grafts^28^,^29^,^30^,^31^,^32^,^33^, neuron-derived cells^34^ and hepatocytes^35^,^36^. Recently, thiol-ene chemistry has been applied for the preparation of polyester type polyHIPE materials, which were also successfully applied as scaffolds for fibroblast^37^, keratinocyte^38^ and osteoblast^39^ cell growth. The ability to control scaffold microstructure is a desirable characteristic in cartilage tissue engineering and thus, PHP may represent the solution to control such complex architectures. In addition, PHP allows the customization to fit the patient’s needs and the site of desired application, and it supports osteoblast and bone ingrowth, which is also one of the crucial factors for *in vivo* application and its integration in cartilage repair.

Here, we show that PHP supports chondrogenic differentiation *in vitro*. Furthermore, we have shown that the prepared PHP-chondrocyte scaffolds promote cartilage growth. To the best of our knowledge, this is the first study showing the applicability of PHP scaffolds in cartilage tissue engineering using human derived primary chondrocyte cell cultures. Our PHP scaffolds were prepared, considering all the requirements of an ideal scaffold for cartilage tissue engineering^9^,^10^,^11^,^12^,^13^,^14^,^15^,^16^, in particular, with regards to pore size (in the range of 50–170 μm), porosity (more than 80%), mechanical properties and biodegradability. In the present study we were specifically interested in mimicking the superficial cartilage layer by the developed PHP scaffolds. An important indicator of success was in our case also the depth of cell migration into the scaffold interior after the cells were plated only onto the scaffold surface. The prepared PHP scaffolds were characterized using confocal microscopy and histological analysis to evaluate the cell viability and the depth of cell ingrowth. The PHP-cell interaction and viability was evaluated *in vitro* using a Live/Dead assay. Additionally, mechanical properties and the degradation rate were determined. Finally, immunohistochemistry was performed and cartilage specific gene expression was analyzed in order to prove the materials suitability for cartilage growth.

## Results and Discussion

**Figure 1:**
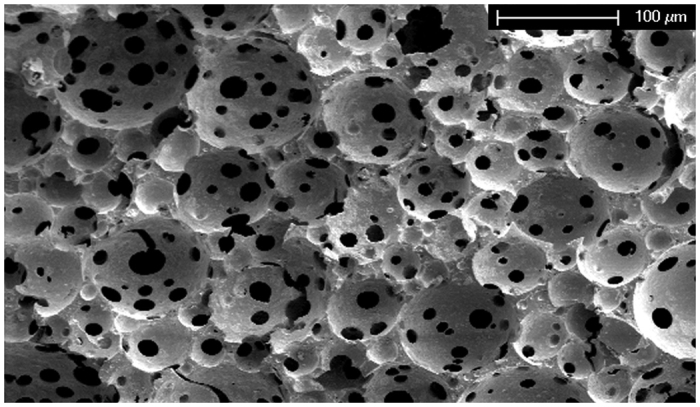
SEM image of a PHP sample (500x magnification).

**Figure 2:**
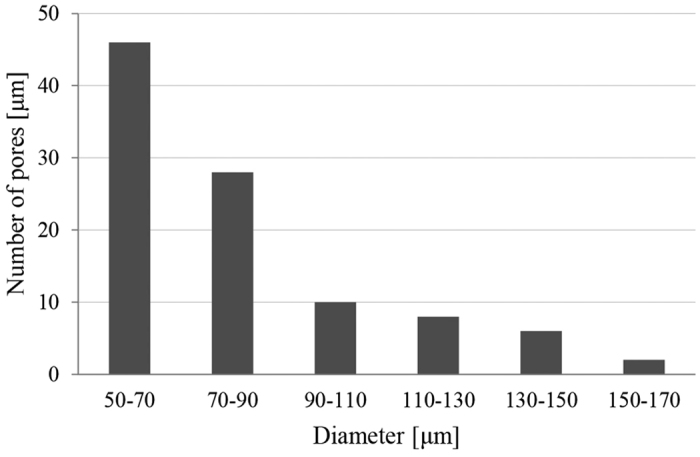
Pore size (diameter of cavities) distribution of PHP.

The polyester type of scaffold was prepared using emulsion templating with a high volume fraction of internal phase in order to achieve high porosity. We focused on the preparation of PHPs based on vinyl esters, which were shown to exhibit no or low cytotoxicity^40^,^41^. As previously shown^29^,^39^, tetrakis-3-mercaptopropionate (TT) and divinyladipate (DVA) have proven appropriate for constructing a polymer network within the emulsion template. Adequate pore size, high degree of porosity and interconnectivity is required for cells to attach, migrate and proliferate, and to allow sufficient nutrition, mass transport, and tissue growth^1^,^2^,^3^,^4^,^5^,^6^,^7^,^8^. Compared to our previous preparation of thiol-ene based PHP material^29^,^39^, an optimized procedure was used for PHP preparation, such as lower stirring rate (50 rpm) with extended mixing time and higher temperature (30 °C), in order to achieve a highly porous interconnected structure with a larger pore size for the purpose of cartilage tissue engineering (see Table 1 for structural and morphological data, Fig. 1 for SEM micrograph of PHP sample). The interconnected porous morphology is the result of the specific preparation procedure, where droplets of the internal phase of emulsion template the primary pores (cavities) while interconnecting pores are formed due to the rupture of the polymer film between the droplets. The resulting primary pores are roughly in the range of 50–170 μm (with more than 70% of pores between 50 and 90 μm), as shown in Fig. 2. The characteristics are comparable to several other materials reported in relation to cartilage tissue engineering^1^,^3^,^4^,^8^. In addition, PHP materials exhibited a range of pore sizes that was suggested to further facilitate chondrocyte activity and appropriate gene expression^6^.

### Degradation of PHP samples

**Figure 3:**
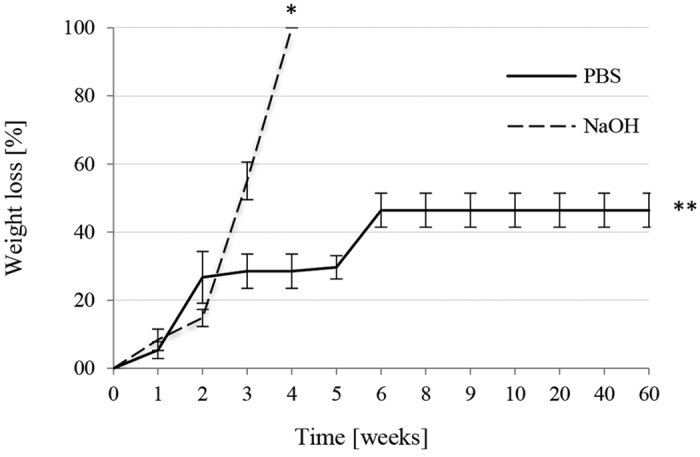
Degradation of PHP in PBS and 0.01 M NaOH. * Full material degradation (4 weeks), ** endpoint.

TT-DVA copolymers are known to be biodegradable via the breakdown of ester bonds^38^. Both a phosphate buffer (PBS) and an accelerated degradation test (NaOH solution) were used to demonstrate the chemical stability of PHP. The results of PHP degradation in both media are shown in Fig. 3. The degradation profile of the PHP samples after exposure to PBS showed approximately 23% of the overall weight loss within 3 weeks and approximately 45% after 6 weeks. Accelerated degradation tests (0.01 M NaOH solution) resulted in weight loss of approximately 55% after three weeks while the sample fully dissolved after 4 weeks. The degradation is probably facilitated by the high porosity and interconnectivity of the PHP structure, hence a large accessible PHP surface for the media. The degradability profile of PHP samples together with the stable mechanical properties indicates a favorable match between degradation rate and new tissue formation. The degradation tests indicate that the PHP material is biodegradable, therefore the material is also suitable for *in vivo* application, wherein the additional enzymatic degradation takes place (e.g. through lipases, hydrolases, etc.)^42^,^43^. The latter was illustrated by the accelerated degradation in the present study in consideration of further PHP adjustments for even more specific applications.

### Mechanical testing

In order to further evaluate the suitability of PHP samples for cartilage scaffolds, they were tested for mechanical properties by dynamic mechanical analysis (DMA) using conditions, resembling the target physiological environment in the articular joint^44^,^45^. In the present study the parameters were adjusted to conditions similar to walking^44^. The values of the Young’s moduli measured at 1 Hz and at 37 °C were 0.15 ± 0.01 MPa for PHP sample and 0.18 ± 0.01 MPa for PHP-chondrocyte constructs after 20 days of cell culturing. According to a report, these values correspond to 38% and 45% of the human articular cartilage Young’s modulus, respectively (0.4–0.8 MPa)^46^,^47^. For comparison, the lower value was chosen; since walking conditions (1 Hz) are connected with a lower Young’s modulus^44^. This in turn indicates that the mechanical properties of the PHP scaffold were adequate to support the proliferation of cells and cartilage growth.

### Morphological analysis of PHP-chondrocyte constructs

#### Confocal microscopy of PHP-chondrocyte constructs

**Figure 4:**
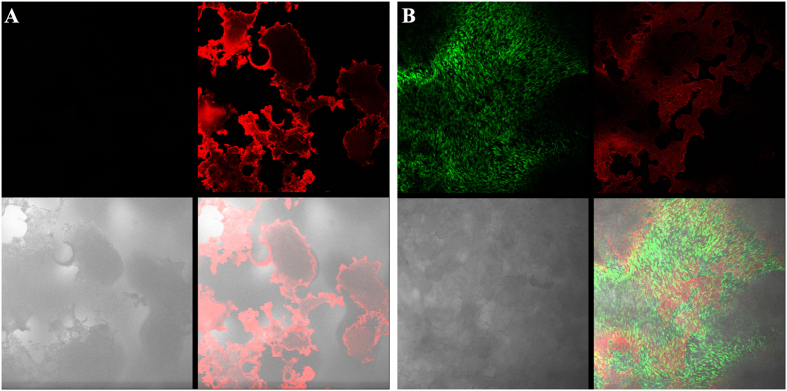
Confocal microscopy of PHP sample and PHP-chondrocyte constructs. Micrographs taken with the confocal microscope: A) as-prepared PHP sample (without cells) after live/dead staining, and B) PHP-chondrocyte construct after cell seeding (7 days) and live/dead staining. Upper parts of both images are the green and red channels, respectively, while the bottom left part shows the bright field image and the bottom right part, the combined image of all channels.

The cell viability on PHP was confirmed with Live/Dead assay, followed by confocal microscopy. The test was performed after 7 days of cell culturing. Figure 4 shows the micrographs of the PHP prior (A) and after (B) cell seeding. As can be seen from Fig. 4B in green, living cells are forming a multilayered cell film on the PHP surface. Since we observed intensive red light on the sample surface as well (Fig. 4B – right top part), we stained the pure (as-prepared) PHP sample (without cells) as a negative control. Figure 4A shows the result of this procedure, suggesting that the observed red fluorescence results from the material and not from dead cells. Additionally, in Fig. 4B no structure within the cell layer was observed in red, which again suggests the absence of dead cells. The background red fluorescence resulting from the material was subtracted from the combined image of all channels to detect any potential dead cell. However, the absence of dead cells may be due to the average lifespan of chondrocytes that exceeds one week, which is more than the time of duration of our experiments^48^. Since only viable cells were observed by Live/Dead assay no additional quantitative viability assessment was performed. Hence, PHP material was proven biocompatible with human articular cartilage and thus appropriate for chondrocyte culturing.

#### SEM analysis of PHP-chondrocyte constructs

**Figure 5:**
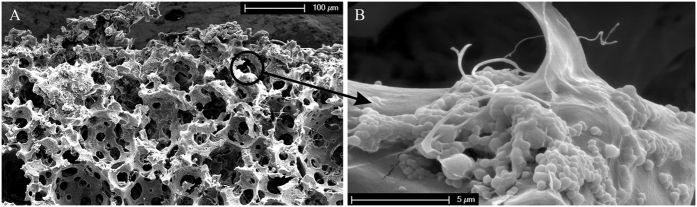
SEM micrographs of PHP-chondrocyte construct after 20 days of cell culturing; (**A**) at low magnification (500x), and (**B**) at high magnification showing cell debris (15000x).

The morphology of PHP-chondrocyte constructs was further evaluated using SEM after 10 and 20 days of cell culturing. In Fig. 5 we show the PHP-chondrocyte construct after 20 days of cell culturing. Micrographs were taken on the surface of PHP-chondrocyte constructs as well as at the cracks that were formed spontaneously during breakage in liquid nitrogen. A range of SEM imaging in different scaffold regions was performed to detect any changes in morphology of the scaffold at different cultivation times. The results showed a similar architectural structure compared to the PHP sample (without cells) (Fig. 1) with recognizable pores and interconnections indicating stability of the material (Fig. 5A). Hence, the ability of the scaffold to maintain its original architecture characteristics in response to the biological material was confirmed. This was also supported by stable mechanical properties after 20 days of cell culturing and a controllable degradation rate. As expected, cells were damaged during the preparation procedure, as well as during the imaging process itself (Fig. 5B). The observed morphology of the cell debris is in agreement with literature refs 49, 50, 51 Combined results from both types of microscopy therefore confirm that cells are indeed attached to the surface of the PHP samples.

#### Histological evaluation

**Figure 6:**
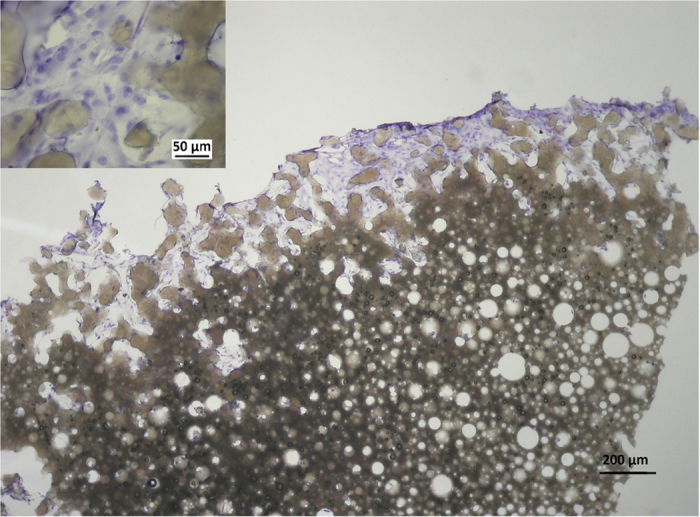
Histological specimen of a PHP-chondrocyte construct after 16 days of culturing and HE staining at low (40x) and high magnification (400x). The micrograph shows 20 μm thick central slice of approximately 2 mm thick PHP piece.

**Figure 7:**
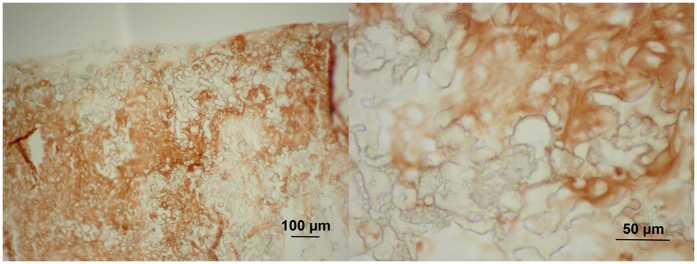
Immunohistochemistry for collagen type 2 of a PHP-chondrocyte construct after 16 days of culturing at low (100x; left image) and high magnification (400x).

The samples for histological evaluation were prepared from PHP-chondrocyte constructs for HE and immunohistochemistry for collagen type 2 after 16 days of cell culturing. Figure 6 shows the photomicrographs of the PHP-chondrocyte construct after HE staining. Viable cells with round nuclei filled the scaffold pores and cells with small nucleoli were also present. Immunohistochemistry (Fig. 7) showed abundant accumulation of collagen type 2 produced by chondrocytes in the newly-formed PHP constructs. Histological evaluation once more confirmed the multilayered cell organization on the as-prepared PHP-chondrocyte constructs as was observed by confocal microscopy and SEM.

A further objective of this study was the demonstration of cellular migration and ingrowth into the prepared PHP materials. Although, chondrocytes form multicellular layers on the PHP scaffold surface more intensely than penetrate the matrix interior, they were nevertheless shown to migrate (or grow into) approximately 300 μm into the scaffold after 16 days of culturing as demonstrated in histological specimens (Fig. 6). Similar penetration depth after the same time of exposure was reported for PHP materials with different cell types^28^. The resulting slower transport deeper into the matrix interior is most probably connected to the material morphological characteristics, e.g. small interconnecting pore size (6–19 μm), but can also be the consequence of a limited nutrient transport. However, the histological studies showed favorable environment for chondrocytes growth on PHP samples with accumulation of collagen type 2 and the resulting migration depth was in accordance with the thickness of superficial cartilage layer in the knee joint^52^,^53^.

## Molecular Analysis

**Figure 8:**
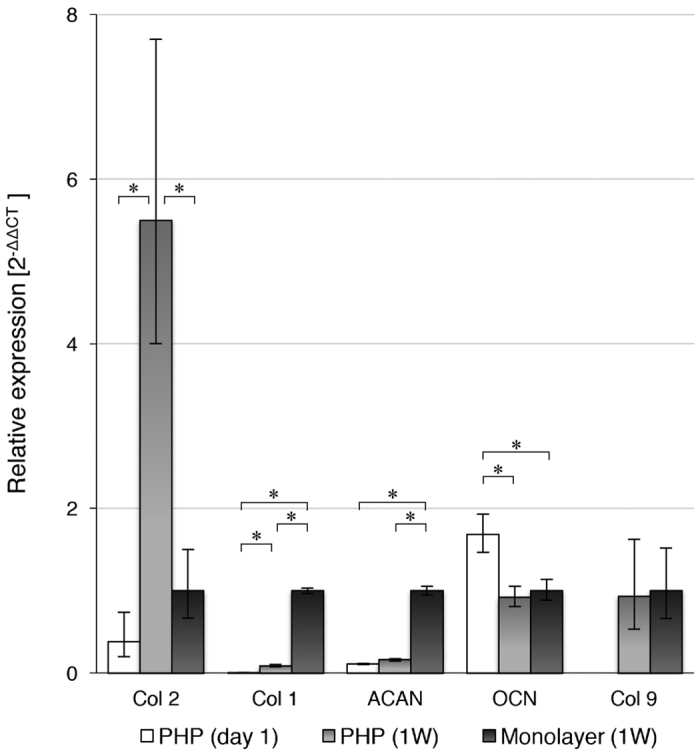
Molecular analysis of collagen type 2 (Col 2), collagen type 1 (Col 1), aggrecan (ACAN), osteocalcin (OCN) and Collagen type 9 (Col 9) on PHP-chondrocyte constructs and monolayer after 7 days of cell culturing. Quantitative analysis of Col 2, Col 1, ACAN, OCN and Col 9 gene expression levels were normalized with the respect to the expression level of house-keeping gene (GADPH) and are presented as 2^−ΔΔCt^ expressions relative to the control (monolayer culturing) ± standard error of expression. Statistical significance (*) was determined at P 0.05, (W) - week.

Molecular analysis was performed in chondrocytes grown on PHP-chondrocyte constructs to investigate the expression of cartilage-specific genes and to monitor phenotype differentiation. The analyzed genes (collagen type 1, 2 and 9, aggrecan and osteocalcin) were chosen according to the literature to monitor cartilage phenotype alterations^17^,^18^,^19^,^54^,^55^,^56^. The results of the analyzed genes on PHP-chondrocyte constructs at a starting point (day 1) and after 7 days of culturing are presented relative to the control (chondrocytes on monolayer) in Fig. 8. A control sample was used to monitor the potential of chondrogenic differentiation on PHP samples after total confluence was reached at 7 days. The housekeeping gene (GADPH) was used as an internal control for RNA sample loading.

Significant up-regulation of collagen type 2 gene expression between first and 7^th^ day of culturing on PHP-chondrocyte constructs (16 fold) and compared to the control sample (chondrocytes on monolayer) (5 fold) indicates that PHP samples may provide a suitable environment to promote cartilage phenotype. This was also confirmed using immunohistochemistry (Fig. 8). Similarly, the retained level of aggrecan gene expression on PHP-chondrocyte constructs also demonstrates the ability of PHP to preserve primary chondrocyte phenotype. Collagen type 1 gene expression, which is unspecific for cartilage ECM and a common sign of dedifferentiation towards fibroblastic phenotype, was significantly increased from day 1 on PHP samples compared to monolayer after 7 days of culturing (500 fold) and to a lesser extent also on PHP after 7 days (43 fold). Despite the increase of collagen type 1 the overall collagen type 2 to collagen type 1 ratio was still favorable in PHP samples after 7 days of exposure. In addition, cartilage phenotype was further promoted with the up-regulation of cartilage specific collagen type 9 which was not detected after the first day and down-regulation of osteogenic marker osteocalcin on PHP chondrocyte cultures after 7 days of culturing. Furthermore, substantial increase in total mRNA content was observed during the culturing on PHP-chondrocytes constructs which also confirms the favorable environment for cell growth.

On the other hand, significant down-regulation of aggrecan (9.3 fold) on PHP chondrocyte construct compared to monolayer culturing after 7 days may suggest that the chondrocytes were undergoing a phenotypic shift in the response to the material. Nevertheless, the presence of aggrecan gene expression in equal amounts during the 7 days culturing period on PHP-chondrocyte constructs, indicates the potential of the PHP architecture to preserve primary chondrocyte phenotype. Hence, PHP microstructure can be further optimized to promote a production of bigger molecules such as proteoglycans. It is also possible that the cells were developing characteristics associated with the superficial zones of articular cartilage, where collagen fibers are typically present (primarily type 2), whereas proteoglycans are more abundant in deeper zones^53^,^57^. Accordingly, chondrocytes were spread over the PHP surface in multicellular layers (as confirmed using confocal microscopy and histology) with a relatively high number of flattened chondrocytes, which are also typically present in superficial cartilage zones. The latter is also in agreement with immunohistochemistry and the determined mechanical properties of the prepared PHP-chondrocyte constructs.

## Conclusion

For the first time, a polyHIPE polymer-based scaffold with polyester chemistry has been used to promote human derived chondrocyte proliferation and cartilage growth. The scaffold exhibits desired biocompatibility and preserves the chondrocyte phenotype. There was no sign of materials’ cytotoxic effects, as evaluated by using commonly employed analysis methods. Furthermore, histological analysis showed viable cells with round nuclei, which filled the scaffolds’ surface pores, and formed a several hundred micrometers thick multilayered film on the surface with accumulation of collagen type 2. In addition, favorable mechanical properties and degradability of PHP samples and PHP-chondrocyte constructs add to the materials’ applicability for cartilage tissue engineering, confirmed by *in vitro* testing on human derived chondrocytes. The flexibility of the polyHIPE preparation procedure allows further optimization of morphology towards an improved artificial cartilage scaffold.

## Methods

### PHP preparation

PHP was prepared according to a published procedure^29^,^39^. Briefly, monomers divinyladipate (DVA, Sigma-Aldrich) and tetrakis-3-mercaptopropionate (TT, Sigma-Aldrich) were used, with a ratio of thiol to vinyl groups 1:1. The aqueous phase (1% solution of CaCl_2_) was added dropwise to the oil phase consisting of monomers, surfactant (poly(ethylene glycol)-block-poly(propylene glycol)-block-poly(ethylene glycol) (Pluronic L121, Sigma Aldrich) and initiator, with enough aqueous phase added to produce a high internal phase emulsion with 85 vol% aqueous phase. Emulsions were transferred to silicone molds and polymerized in a UV chamber (Intelliray 600, Uvitron, USA) at 80% intensity for 140 s. The polymers were purified by Soxhlet extraction in water for 24 h and in ethanol for 24 h.

#### Determination of morphology, pore size and interconnected size distribution

All PHP samples were investigated with a field emission scanning electron microscope (SEM; Quanta 200 3D, FEI Company, USA) operated at 10 kV to 15 kV. The SEM micrographs of PHP samples were used to determine pore and interconnecting pore size. Prior to analysis, the samples were immersed in liquid nitrogen, broken down to an appropriate size (approx. 4 mm in length) and gold coated. The average pore diameters were determined from SEM micrographs by measuring at least 50 pores per micrograph, using a correction factor 2/√3 to compensate for the random sectioning of the pores^23^. Tree independent PHP samples were used for the characterization. The results are presented as the mean ± standard deviation (SD).

#### Degradation tests

Materials were degraded in different degradation media, chosen to simulate the conditions, similar to the desired final application. The samples with a weight of approximately 3 g were placed in 50 ml of phosphate buffer saline (PBS) at 37 °C and were weighed once per week. Prior to weighing, samples were washed with deionized water, ethanol and dried until constant mass. The same procedure was performed for the degradation in 0.01 M NaOH solution. All degradation tests were performed in triplicates.

### Cell isolation, cell seeding, and *in vitro* culturing

#### Isolation of primary chondrocytes

Full-thickness cartilage was surgically removed from the femoral condyle of arthritic knee of a 50 years old patient who underwent total knee arthroplasty performed at University Clinical Center in Maribor, Slovenia. Prior to surgery, no systemic disease or any treatment was reported for the included patient. The study was conducted in accordance with the *Declaration of Helsinki* and its subsequent amendments and was approved by the National Ethics Committee (Ljubljana, Slovenia). The patients’ informed consent was obtained.

The cartilage was dissected from subchondral bone, sliced into small pieces, digested with 0.25% Trypsin/EDTA (Sigma, France) for 3 hours at 37 °C and 5% CO_2_, centrifuged at 1500 rpm for 5 minutes, rinsed with culturing medium - Advanced DMEM (Invitrogen, Germany) that was supplemented with Penicillin 100 IU/ml, Streptomycin 1 mg/ml, L-glutamine 2 mM and 5% fetal bovine serum (FBS) and plated on 25 cm^2^ flasks. The primary chondrocytes were then left until confluence was reached. The culturing medium was changed every three days. Chondrocytes were counted with a hemocytometer using trypan blue staining. Only cells from passage 2 were used for the conducted experiments.

#### Cell seeding onto PHP samples

The PHP samples were prepared in the form of oval disks (diameter around 7 mm and thickness around 2 mm). Prior to cell seeding, samples were autoclaved and later immersed in the culture medium overnight. SEM control was performed to ensure that the PHP structure remained unaffected by the autoclaving process. The medium was removed and approximately 1 × 10^6^ cells were applied onto each PHP sample. The as-prepared PHP-chondrocyte constructs were incubated for 4 hours, followed by addition of culturing medium -Advanced DMEM supplemented with 5% FBS and 50 ng/ml TGF-beta1, which was changed every three days. The addition of growth factor was performed according to the literature^17^,^18^. All the cultivation was done at 37 °C in a humidified atmosphere with 5% CO_2_. Cell cultivation on PHP samples was performed for a total of 20 days. Analysis using the Live/Dead Kit (Sigma-Aldrich, Germany) was performed after 7 days of culturing, SEM and molecular analysis after 10 and 20 days, histology after 16 days and mechanical testing after 20 days. Three samples were used to perform independent sets of experiments and average values are reported if not stated otherwise. Monolayer cell cultivation for 7 days served as a control to monitor the potential of chondrogenic differentiation on PHP samples. Monolayer cell culturing was held under the same culturing conditions until total confluence was reached, which was observed after 7 days of culturing.

### Mechanical testing

In order to evaluate the material’s mechanical properties as a function of temperature, dynamic mechanical analysis (DMA) was performed on the PHP samples and PHP-chondrocyte constructs after 20 days of culturing. DMA was assessed on the Dynamic mechanical analyzer DMA 8000 (Perkin Elmer, USA). Prior to measurements PHP samples and PHP-chondrocyte constructs (after 20 days of culturing) were immersed in 3% formaldehyde and dried till constant mass. For the measurement, film samples of PHP were prepared conforming to the dimensional limits of the tension film clamp test fixture (5 mm wide and 10 mm in length). The DMA test procedure was as follows: after mounting the film sample in the DMA tension film clamp, the temperature was adjusted to 25 °C. The sample was then heated at 0.5 °C/min until 40 °C. The samples were tested, using a dynamic force with a single frequency oscillation of 1 Hz and amplitude of 0.005 mm. The storage modulus for each run was calculated as a function of increasing temperature, using thermal analysis software. All mechanical testing was performed in triplicates with at least five independent measurements for the single temperature. The results are presented as the mean ± standard deviation (SD).

### Morphological analysis of PHP-chondrocyte constructs

#### Live/dead kit and confocal microscopy

The viability of chondrocytes on and within the PHP-chondrocyte constructs was assessed using the Live-Dead assay. As described previously^14^ the specimen were rinsed with PBS, incubated in 4 mM calcein AM and 2 mM ethidium homodimer in PBS for 30 minutes at 37 °C, and finally washed with PBS. Subsequently, the constructs were observed under a confocal microscope (SP5 AOBS, Leica Microsystems, Germany). Viable cells were detected by the presence of intracellular esterase activity, which converts calcein-AM to calcein and produces a green fluorescence. Dead cells were recognized by ethidium homodimer, which enters the cell through the damaged plasma membrane and binds with deoxyribonucleic acid to produce red fluorescence. For a negative control, the PHP material without added cells was also stained and evaluated. The procedure was performed for three respective samples, while the observation using confocal microscopy was performed on three different surface regions on each sample.

#### SEM analysis of PHP-chondrocyte constructs

SEM analysis of PHP-chondrocyte constructs was performed after 10 and 20 days of culturing. Prior to SEM analysis PHP-chondrocytes constructs were immersed in 3% formaldehyde solution for 24 hours and dried using lyophilization for 24 hours. Further analysis procedure was performed as described above. SEM examination was performed on the samples’ surface and at fracture points, produced during the preparation procedure.

#### Histological preparation and analysis

After 16 days of cultivation PHP-chondrocyte constructs were washed in PBS, fixated in 4% PFA, embedded in OCT (Tissue-Tek, Sakura Finetek Europe B.V., The Netherlands) and sectioned at 20 μm with a Leica cryostat. Sections werestained with hematoxylin-eosin (HE), analyzed and recorded with bright field microscope (Nikon Eclipse 200). The cellular migration was also estimated from the histological specimen and was defined as the depth at which the majority of cells were still present. The histological analysis was performed on two independent sections on multiple samples.

#### Immunostaining of collagen type 2

Collagen 2 immunohistochemistry was performed as previously described^58^ using mouse monoclonal anti-collagen 2 antibody (II-II6B3; DSHB Iowa) with Dako EnVision detection kit. In brief, slides were treated with 0.1% pronase (P-8811; Sigma) in PBS for 20 minutes at room temperature, followed by 2.5% hyaluronidase (H-3506, Sigma) in PBS for 30 minutes at 37 °C. Endogenous peroxidase activity was quenched with 3% H_2_O_2_ for 10 minutes at room temperature. Nonspecific binding sites were blocked with 10% goat serum in PBS for 60 minutes at room temperature. Sections were then incubated with primary antibody against collagen type 2 diluted 1:50 in PBS overnight at 4 °C. The slides were then washed in PBS, and incubated with the secondary antibody (Dako Real Envision/HRP, K5007) for 30 min at room temperature. DAB (Dako Real DAB+Chromogen, K5007) was applied for about 2 min and then removed by rinsing with distilled water. Slides were mounted with Biomount Aqua (Biognost, Croatia) and covered. Exclusion of primary antibody resulted in no staining.

### Molecular analysis

#### Gene expression analysis (RNA extraction, quality and transcription into cDNA)

After 1^st^ and 7^th^ day of incubation, the PHP-chondrocyte constructs were transferred to micro centrifuge tubes, 1.4 mL of TRI reagent (Sigma, Steinheim, Germany) was added and the tubes were vigorously vortexed for 30 min at room temperature. After 30 min, 280 μL of chloroform (Sigma, Germany) was added, the tubes were further vortexed for 15 min and centrifuged at 12.000 × g at 4 °C for 15 min. Further extraction process and extraction from cell monolayers was carried out according to the manufacturer’s instructions (Sigma, Steinheim, Germany). Concentration and purity of extracted cellular RNA was measured using NanoDrop 2000c (Thermo Scientific, Delaware, USA) optical density readings at 260 nm and 260/280 nm ratio, respectively. RNA was transcribed into cDNA using cDNA reverse transcription kit (Applied Biosystems, California, USA). 2 μL of each cDNA sample with concentration of 15 ng/μL was used for qPCR analysis.

#### Primer sequences

Primer sequences for target genes aggrecan (ACAN), collagen type 2 (Col 2), type 9 (Col 9), osteocalcin (OCN) and reference gene glyceraldehyde-3-phosphate dehydrogenase (GAPDH) were obtained from Caterson *et al*.^56^ and corresponding mRNA sequences were retrieved from PubMed Nucleotide database (www.ncbi.nlm.nih.gov/nuccore/) and AceView database^59^. Primers for the target gene collagen type 1 (Col 1) were designed using IDT oligo analyzer (eu.idtdna.com/calc/analyzer). Primer sequences and corresponding mRNA sequence NCBI accession numbers are listed in Table 2. The expression of the house-keeping gene (GADPH), was used as an internal control to normalize for the difference for RNA concentration and loading of samples.

#### Quantitative real time PCR analysis

Quantitative real time PCR was performed using LightCycler 480 thermocycler (Roche, Basel, Germany) and with 2 × Maxima SYBR Green qPCR master mix (Life Technologies, California, USA) according to the manufacturer’s instructions. PCR reactions were carried out in a volume of 10 μL. The thermal profile of qPCR assay was: initial denaturation at 95 °C for 10 min, denaturation at 95 °C for 15 s, annealing at 60 °C for 30 s and extension at 72 °C for 30 s, 40 cycles. Melting curves were obtained at the end of assay run with continuous acquisition at 0.11 °C/s from 65 °C to 95 °C. Before qPCR assays were performed, the quality and specificity of PCR amplicons were checked using end-point PCR and agarose gel electrophoresis. After each PCR assay, melting curve analyses were also performed in order to confirm specific amplifications. qPCR data was normalized and calculated with 2^−ΔΔCt^ method as previously described^60^.

#### Statistical analysis

All shown experiments were performed in triplicates. Statistical analysis was performed using IBM SPSS Statistics 22 (IBM Inc., New York, USA) with Mann-Whitney U test. Gene expression data for Col 1, Col 2, Col 9, ACAN and OCN were analyzed in linear manner using 2^−ΔCt^ and were calculated as 2^−ΔΔCt^ expressions relative to the control (monolayer culturing) ± standard error of expression.

## Additional Information

**How to cite this article**: Naranda, J. *et al*. Polyester type polyHIPE scaffolds with an interconnected porous structure for cartilage regeneration. *Sci. Rep.* **6**, 28695; doi: 10.1038/srep28695 (2016).

## Figures and Tables

**Table 1:** Characteristics of the fabricated polyHIPE scaffolds.

Parameter	Values
Sample dimensions	Round disks:
	- diameter: ≈7 ± 0.1 mm
	- thickness: ≈2 ± 0.1 mm
Pore diameter	Mean ± SD: 82 ± 26 μm
	Range: 50–170 μm
Interconnecting pore diameter	Mean ± SD: 13 ± 3 μm
	Range: 6–19 μm
Porosity	85%

*SD = standard deviation.

**Table 2:** Primer sequences of target and reference genes.

Gene	Gene name	Accession number	Primer sequence 5′ → 3′
*Col 2*	Collagen type 2, alpha 1	NM_001844.4 NM_033150.2	TTTCCCAGGTCAAGATGGTC CTGCAGCACCTGTCTCACCA
*Col 1*	Collagen type 1, alpha 1	NM_000088.3	CGGCTCCTGCTCCTCTTAGCACACGTCTCGGTCATGGTA
*ACAN*	Aggrecan	NM_013227.3 NM_001135.3	TGAGGAGGGCTGGAACAAGTACCGGAGGTGGTAATTGCAGGGAACA
*OCN*	Osteocalcin; Bone gamma-carboxyglutamate (gla) protein	NM_199173.4	ATGAGAGCCCTCACACTCCTCGCCGTAGAAGCGCCGATAGGC
*Col 9*	Collagen, Type IX, alpha 1	NM_001851.4	GTGTTGCTGGTGAAAAGGGTGGGATCCCACTGGTCCTAAT
*GAPDH*	Glyceraldehyde-3-phosphate dehydrogenase	NM_001289745.1 NM_002046.5 NM_001289746.1 NM_001256799.2	GGGCTGCTTTTAACTCTGGTTGGCAGGTTTTTCTAGACGG
